# FliA regulates the antibacterial activity of plantaricin BM-1 against *Escherichia coli* K-12 through the LuxS/AI-2 quorum-sensing-mediated biofilm formation

**DOI:** 10.3389/fmicb.2025.1606567

**Published:** 2025-06-20

**Authors:** Shichun Wang, Xiaodong Song, Xuan Zheng, Congyang Cheng, Junhua Jin, Yuanhong Xie, Hongxing Zhang

**Affiliations:** ^1^Beijing Laboratory of Food Quality and Safety, Beijing Key Laboratory of Agricultural Product Detection and Control of Spoilage Organisms and Pesticide Residue, College of Food Science and Engineering, Beijing University of Agriculture, Beijing, China; ^2^Key Laboratory of Dairy Quality Digital Intelligence Monitoring Technology, State Administration for Market Regulation, Inner Mongolia Mengniu Dairy (Group) Co., Ltd., Hohhot, China

**Keywords:** class IIa bacteriocins, *Escherichia coli*, FliA, biofilm formation, transcriptome

## Abstract

**Introduction:**

Plantaricin BM-1 is a class IIa bacteriocin active against *Escherichia coli*. However, the mode of action of class IIa bacteriocins against gram-negative bacteria remains unclear. In this study, the regulatory role of sigma factor FliA (σ^28^) in the antibacterial mechanism of plantaricin BM-1 against *E. coli* K-12 BW25113 is evaluated.

**Methods:**

The *fliA*-complemented strain of *E. coli* JW1907, namely *E. coli* ReJW1907, was constructed through λ-Red homologous recombination. The effects of plantaricin BM-1 on *E. coli* growth, cell morphology, and membrane integrity were investigated using growth curves, electron microscopy, and flow cytometry. The biofilm formation ability of *E. coli* was evaluated using crystal violet staining and confocal laser scanning microscopy. Transcriptomic analysis was performed to screen for differentially expressed genes (DEGs).

**Results and discussion:**

The inhibition rate (I%) of plantaricin BM-1 (3.75 mg/mL) against *E. coli* JW1907 (89.22 ± 1.13%) at the 8th h of culture was significantly higher than that of *E. coli* BW25113 (70.36 ± 6.30%) and ReJW1907 (74.75 ± 4.99%). The biofilm biomass produced by *E. coli* BW25113 (OD_595__*nm*_ = 0.343 ± 0.056) was significantly reduced to 0.227 ± 0.04 after *fliA* deletion, and was recovered to its original level (0.358 ± 0.027) after *fliA* complement. A total of 205 DEGs were identified between *E. coli* BW25113 and JW1907. Among these, four DEGs (*fliZ*,*wza*, *lsrR*, and *pgaA*) were enriched in the biofilm formation pathway. Further analysis revealed eight up-regulated DEGs (*lsrKRBDCAFG*), which were significantly enriched in the LuxS/AI-2 quorum sensing (QS) system. After the deletion of any gene from *lsrKRBDCAFG*, the I% of plantaricin BM-1 against *E. coli* BW25113 (70.77 ± 7.01%) was significantly increased to 80.68–90.06%, with its biofilm production (0.254 ± 0.014) reduced to 0.135–0.188. In conclusion, FliA modulates biofilm formation through the LuxS/AI-2 QS system, thereby regulating the antibacterial activity of plantaricin BM-1. Overall, these findings improve our understanding of the bacteriostatic mechanism of class IIa bacteriocins against gram-negative bacteria.

## 1 Introduction

Bacteriocins are small antimicrobial peptides synthesized ribosomally by various bacteria (gram-positive and gram-negative) and archaeal species (Fathizadeh et al., 2022). Most bacteriocins exhibit antagonistic activity against bacteria closely related to their producer strains, whereas a few others have a relatively broad antibacterial spectrum (Yang et al., 2014). Currently, bacteriocins produced by gram-positive lactic acid bacteria (LAB) have gained significant attention in the food industry (Fernandes and Jobby, 2022) owing to their proven safety for human health and the environment, potent antibacterial activity against various food-borne pathogenic bacteria, even at trace concentrations, and simpler synthesis methods that do not require multi-enzyme systems (Cotter et al., 2013). Their unique mode of action reduces the risk of microbial resistance compared to traditional antibiotics (Johnson et al., 2018). Specifically, nisin and pediocin PA-1 have been commercialized as food biopreservatives (Souza et al., 2022; Anumudu et al., 2021).

To date, the classification method for bacteriocins remains under debate, with no fixed standard. Based on the molecular mass and structure, amino acid modifications, and physicochemical properties, Klaenhammer (1993) initially classified bacteriocins produced by LAB into four classes (I–IV): (i) class I bacteriocins (also known as lantibiotics) are small peptides (<5 kDa) of 19–50 residues containing non-coding amino acids, such as lanthionine, β-methyllanthionine, dehydrobutyrine, and dehydroalanine; (ii) class II bacteriocins are thermostable unmodified peptides further divided into IIa, IIb, and IIc subgroups; (iii) class III bacteriocins are heat-labile large peptides (> 30 kDa) with relatively narrow antibacterial spectra; (iv) the former class IV bacteriocins are large complexes comprising protein and other macromolecules (lipids and carbohydrates). Notably, class IV members have recently been reclassified as bacteriolysins (Mokoena, 2017).

Class IIa bacteriocins, or pediocin-like bacteriocins, are the most extensively studied and largest subgroup of class II bacteriocins (Ennahar et al., 2000). Over 30 class IIa bacteriocins have been characterized in LAB (Yi et al., 2022), including pediocin PA-1, sakacin P, and enterocin P. These thermotolerant peptides generally consist of 37–48 amino acids and contain a conserved motif, YGNGV/YGNGL, at their N-terminus (Drider et al., 2006). Bacteriocins in this subgroup are regarded as strong inhibitors of *Listeria* species (Tresnak and Hackel, 2020). To date, the molecular mechanisms of action of class IIa bacteriocins against *Listeria monocytogenes* and several other gram-positive bacteria have been elucidated. Specifically, they interact directly with the mannose phosphotransferase system (Man-PTS) IIC and IID subunits in target cells (Diep et al., 2007), forming pores on their plasma membrane (Nissen-Meyer et al., 2009) and disrupting the transmembrane proton gradient (ΔpH) and electric potential (Δψ) (Yi et al., 2022), two parameters of the proton motive force (PMF) crucial for bacterial survival (Farha et al., 2013). Based on the phylogenetic analysis of IIC/IID subunits, Man-PTS can be classified into three groups, and only members of group I (derived from *L. monocytogenes*, *L. innocua*, *E. faecalis*, etc.) can serve as receptors for class IIa bacteriocins (Kjos et al., 2009). Therefore, class IIa bacteriocins are generally considered inactive against gram-negative bacteria (Chalón et al., 2012). However, exceptions contradict this hypothesis. For instance, plantaricin IIA-1A5 isolated from *Lactobacillus plantarum* exhibits antibacterial activity against *Escherichia coli* ATCC 25922 (Mutmainna et al., 2021); bacteriocin E 50-52 shows broad-spectrum activity against *E. coli* O157:H7, *Salmonella* spp., and *Shigella* spp. (Svetoch et al., 2008). However, these studies have focused primarily on the antibacterial activity of class IIa bacteriocins against gram-negative bacteria, with little research being conducted on the underlying mechanisms.

Biofilms are surface-attached communities formed by single or multiple microbial species in a matrix of extracellular polymeric substances, including polysaccharides, proteins, and DNA (Karygianni et al., 2022; Flemming et al., 2023). Biofilms can effectively protect microorganisms from adverse stress and are the primary contributors to bacterial pathogenicity and antibiotic resistance (Sharma et al., 2019). Several studies have reported the anti-biofilm activity of LAB bacteriocins against *E. coli*. Qiao et al. (2021) discovered that bacteriocin BM173 effectively inhibits biofilm synthesis by *E. coli* ATCC 25922 and *Staphylococcus aureus* ATCC 25923. Notably, bacteriocin DF01 disrupts biofilm formation by *E. coli* KCTC 1039 without affecting the established biofilm structure (Kim et al., 2019). Similar anti-biofilm activity against *E. coli* has been reported for the bacteriocins BM1157 (Luo et al., 2020) and LFX01 (Xin et al., 2023).

Quorum sensing (QS) is a population density-dependent intercellular communication system that modulates several metabolic pathways, including biofilm formation, cell motility, and virulence factor production, through autoinducers (AIs), which are extracellular signaling molecules (Mayer et al., 2023). *E. coli* strains produce autoinducer-2 (AI-2) through S-ribosylhomocysteine lyase (LuxS) (Torres-Cerna et al., 2019). The LuxS/AI-2 mediated QS system in *E. coli* has been well characterized, comprising a two-component signaling system (TCS) and several proteins encoded by genes activated through the *lsr* operon (*lsrBDCAFG* and *lsrRK*) (Jani et al., 2017). Briefly, AI-2 is internalized by *E. coli* cells through the ATP-binding cassette (ABC) transporter LsrBDCA and phosphorylated by LsrK kinase. Phosphorylated AI-2 can induce the transcription of several downstream genes. In addition, it binds to and inactivates the *lsr* operon transcriptional repressor LsrR, inducing the expression of the *lsr* genes (Xavier and Bassler, 2005). Finally, phosphorylated AI-2 is degraded by LsrF and LsrG (Marques et al., 2014; Marques et al., 2011).

FliA is an RNA polymerase sigma factor (σ^28^) that activates all class III promoters in *E. coli* (Liu and Matsumura, 1995). The flagellar transcription network consists of several promoters that can be divided into three classes (I–III) based on their chronological order and mode of regulation (Fitzgerald et al., 2014). The transcription factor FlhDC (FlhD_4_C_2_) is regarded as the master regulator of flagellar synthesis, whereas *flhDC* is considered the sole class I operon (Soutourina and Bertin, 2003). FlhDC initiates transcription of all class II operons, including *fliAZY* (Fitzgerald et al., 2014; Soutourina and Bertin, 2003). FliA activates all class III promoters that encode genes associated with late-period flagellar assembly (such as flagella filaments and motors) and bacterial chemotaxis, and regulates the expression of several mRNAs involved in aerotaxis (*aer*) and the cyclic-di-GMP pathway (*yhjH*, *ycgR*) (Soutourina and Bertin, 2003). In addition, several flagellar genes (including *fliA*) in class II operons are regulated by FliA and FlhDC (Soutourina and Bertin, 2003). The connection between FliA and the LuxS/AI-2 QS system has been reported by previous studies (Kim et al., 2010; Sperandio et al., 2001), namely that *fliA* expression is downregulated in enterohemorrhagic *E. coli* O157:H7 after *luxS* deletion. However, the specific mechanism behind this connection has not yet been clarified.

Plantaricin BM-1, a typical class IIa bacteriocin derived from *L. plantarum* BM-1 (Zhang et al., 2013), was previously shown to exhibit antibacterial and anti-biofilm activities against *E. coli* K-12 strain BW25113 (Wang et al., 2021). In an earlier study, we discovered that a PotF (putrescine-binding protein) null mutation in *E. coli* K-12 enhanced the antibacterial effect of plantaricin BM-1 and upregulated FliA expression by 1.74-fold (Wang et al., 2025). Accordingly, we hypothesized that FliA is involved in the antibacterial mechanism of plantaricin BM-1 against *E. coli* K-12. In the present study, we tested this hypothesis and investigated the regulatory role of FliA to improve our understanding of the mode of action of class IIa bacteriocins against gram-negative bacteria, thereby providing a theoretical basis for the wider application of this sort of antimicrobial peptides.

## 2 Materials and methods

### 2.1 Bacterial strains and plasmids

The bacterial strains and plasmids used in this study are listed in Table 1. *E. coli* K-12 strain BW25113 and its derived mutants were cultured in Luria-Bertani (LB) medium. *L. plantarum* BM-1 was cultured in de Man, Rogosa, and Sharpe (MRS) medium. Unless otherwise stated, all bacterial strains were cultured for 12 h at 37°C with a rotational speed of 180 rpm in a horizontal shaker.

**TABLE 1:** Strains and plasmids used for this study.

Strain and plasmid	Genotype/description	Source/references
*E. coli* K-12 strain BW25113	*lacI^q^* *rrnB*_*T14*_ Δ*lacZ*_WJ16_ hsdR514 Δ*araBA*-D_AH33_ Δ*rhaBAD*_LD78_/Parent strain of Keio Collection	(Datsenko and Wanner, 2000; Baba et al., 2006)
*E. coli* JW1907	BW25113 Δ*fliA*::Kan^R^	(Baba et al., 2006)
*E. coli* ReJW1907	JW1907 with *fliA* complemented	This study
*E. coli* JW1509	BW25113 Δ*lsrB*::Kan^R^	(Baba et al., 2006)
*E. coli* JW1508	BW25113 Δ*lsrD*::Kan^R^	(Baba et al., 2006)
*E. coli* JW1507	BW25113 Δ*lsrC*::Kan^R^	(Baba et al., 2006)
*E. coli* JW1506	BW25113 Δ*lsrA*::Kan^R^	(Baba et al., 2006)
*E. coli* JW1511	BW25113 Δ*lsrG*::Kan^R^	(Baba et al., 2006)
*E. coli* JW1510	BW25113 Δ*lsrF*::Kan^R^	(Baba et al., 2006)
*E. coli* JW1505	BW25113 Δ*lsrR*::Kan^R^	(Baba et al., 2006)
*E. coli* JW1504	BW25113 Δ*lsrK*::Kan^R^	(Baba et al., 2006)
*L. plantarum* BM-1	Producer of plantaricin BM-1	Laboratory Preservation
pKD46	Red recombinase plasmid	(Baba et al., 2006)

Kan^R^: Kanamycin resistance gene derived from pKD13 (NCBI Accession No. AY048744)

### 2.2 Preparation of plantaricin BM-1

Plantaricin BM-1 was isolated and purified as described previously (Zhang et al., 2013). *L. plantarum* BM-1 was cultured for 12 h, as described in Section 2.1. Cell-free supernatants (CFS) were collected through centrifugation (9,000 rpm, 15 min) at 4°C. Crude plantaricin BM-1 in the CFS was separated using saturated ammonium sulfate (516 g/L CFS) and centrifuged (9,000 rpm, 15 min, 4°C). Crude plantaricin was dissolved in sterile double-distilled water (ddH_2_O) and purified by dialysis, cation-exchange chromatography, and desalting. The purified plantaricin solution was lyophilized using a FreeZone 6 freeze dryer (Labconco, Corp., Kansas City, MO, United States).

Lyophilized plantaricin BM-1 was re-dissolved in ddH_2_O. The protein concentration of the re-dissolved solution was determined using a NanoDrop 2000 spectrophotometer (Thermo Fisher Scientific, Waltham, MA, United States). Before use, plantaricin BM-1 solution was sterilized by passing through a Millex^®^-GP filter (0.22 μm; Merck Millipore, Germany).

### 2.3 Construction and validation of the *fliA*-complemented transformant

To further investigate the consequences of *fliA* deletion, *fliA*-complemented *E. coli* strain JW1907 was constructed using the lambda red homologous recombination method (Datsenko and Wanner, 2000). *E. coli* BW25113 and JW1907 were cultured for 12 h, as described in section 2.1. Genomic DNA was extracted from *E. coli* BW25113 cultures using a TIANamp bacterial DNA kit (Tiangen Biotech, Beijing, China). The *fliA* fragment (760 bp) was amplified from the *E. coli* BW25113 genome using the fliA-Red-F (5′-AAGTGCGGCATTTACTGACGTTATAACTTACCCAGTTTAG-3′) and fliA-Red-R (5′-TATTGCGTCCCGACAAATAGGTGAATT CACTCTATACCGC-3′) primers and purified through a TIANquick midi purification kit (Tiangen Biotech). Plasmid pKD46 was heat-shocked into *E. coli* JW1907 competent cells, prepared with 0.1 mol/L calcium chloride. Subsequently, 0.01 mol/L L-arabinose was added and incubated with pKD46-harboring *E. coli* JW1907 at 30°C, inducing the full expression of recombinases. The *fliA* fragment was then transformed into *E. coli* JW1907, spread on LB agar plates (containing 50 μg/mL kanamycin), and grown overnight at 37°C. PCR products of *E. coli* BW25113, JW1907, and the positive transformant genomes amplified with the fliA-Red-F/R primer pair were analyzed by 1% agarose gel electrophoresis, and a Takara DL2,000 DNA Marker (Cat# 3427, Takara Bio, Japan) was used. The complementary transformant was renamed *E. coli* ReJW1907.

### 2.4 Antibacterial activity of plantaricin BM-1 against *Escherichia coli*

*E. coli* strains were cultured in LB broth for 12 h. The viable cell concentration in each *E. coli* culture was adjusted to 10^4^ CFU/mL. One milliliter of *E. coli* culture was added to a 48-well plate, and plantaricin BM-1 was added at a final concentration of 3.75 mg/mL, according to our previous study by Chen et al. (2021). The plate was subjected to a MicroScreen real-time microbial growth analysis system (Jieling Instrument Manufacturing Co., Ltd, Tianjin, China) for 12-h at 37°C with a rotational speed of 500 rpm. The optical density at 600 nm (OD_600__nm_) of each well was obtained hourly. *E. coli* strains without plantaricin treatment were used as negative controls, and sterile LB broth was used as the blank control. Three biological replicates were performed for all *E. coli* groups, and the average value was used to plot the bacterial growth curves.

The inhibition rate (I%) of *E. coli* by plantaricin BM-1 was defined as the reduction in bacterial growth according to Luo et al. (2014), and the I% at the indicated point was calculated using the following formula:


$$\mathrm{Inhibition}\,\mathrm{Rate}\,\left(I\%\right)=\frac{\left({\mathrm{OD}}_{\mathrm{CT},t}-{\mathrm{OD}}_{\mathrm{CT},0}\right)-\left({\mathrm{OD}}_{\mathrm{PT},t}-{\mathrm{OD}}_{\mathrm{PT},0}\right)}{\left({\mathrm{OD}}_{\mathrm{CT},t}-{\mathrm{OD}}_{\mathrm{CT},0}\right)}\times 100\%$$

where OD_CT,t_ represents the OD_600__nm_ of untreated *E. coli* at the indicated time point; OD_CT,0_ represents the OD_600__nm_ of untreated *E. coli* at the initial time point; OD_PT,t_ represents the OD_600__nm_ of plantaricin-treated *E. coli* at the indicated time point; OD_PT,0_ represents the OD_600__nm_ of plantaricin-treated *E. coli* at the initial time point; I% = 100% is defined as no bacterial growth; I% > 100% represents bacterial death.

### 2.5 Morphological analysis

Scanning (SEM) and transmission electron microscopy (TEM) were used as previously described (Bian et al., 2022; Chen et al., 2021). *E. coli* BW25113 and JW1907 were incubated with plantaricin BM-1 (3.75 mg/mL) for 12 h at 37°C, as described in section 2.4. The untreated *E. coli* strains were used as controls. *E. coli* cells were collected at 8 and 12 h by centrifugation (8,000 rpm, 4°C, 10 min). After being washed with 0.1 mol/L PBS buffer thrice, *E. coli* cells were fixed with 2.5% glutaraldehyde at 4°C for 24 h. Fixed samples were rinsed with 0.1 M PBS thrice, dehydrated at 25 ± 2°C using a graded ethanol series (30, 50, 70, 80, 90, 95, and 100%), and preserved in 100% ethanol.

For SEM, dehydrated samples were dried using a Quorom k580 critical point dryer (Quorom, UK). The dried samples were coated with conductive carbon blue and fixed on the sample stage; platinum was sprayed using an MC1000 ion sputtering instrument (Hitachi, Japan). Cell samples were analyzed using SEM (Region 8100, Hitachi, Japan).

For TEM, the dehydrated samples were treated twice with 100% acetone for 10 min each and soaked in different proportions of acetone and EMBed 812 resin mixtures in turn (3:1 for 1 h; 1:1 for 2 h; 1:3 for 2 h; pure EMBed 812 for 8 h) at 37°C. The samples were inserted into the embedding models containing pure EMBed 812 resin for polymerization, which was performed for 24 h at 65°C. The polymerized samples were sliced to create 70–90 nm sections using a UC7 ultramicrotome (Leica, Germany). The sections were transferred to copper grids and stained with 2% uranyl ethanol and lead citrate. After being washed with deionized water, the samples were dried overnight at 25 ± 2°C and analyzed using TEM (HT7800, Hitachi, Japan).

### 2.6 Flow cytometric analysis

Flow cytometry was performed to assess the damage caused by plantaricin BM-1 to the cell membrane integrity of *E. coli* cells according to the method described by Li et al. (2013), with several modifications. *E. coli* BW25113 and JW1907 were cultured in LB broth for 12 h. The concentration of viable cells in each *E. coli* culture was adjusted to 10^6^ CFU/mL. Next, 1 mL of each culture sample was transferred to a sterile 1.5-mL Eppendorf tube, and plantaricin BM-1 was added to a final concentration of 3.75 mg/mL and incubated at 37°C for 8 h and 12 h. Untreated *E. coli* cells were the control group. *E. coli* cells were collected by centrifugation (8,000 rpm, 4°C, 10 min), washed with PBS buffer thrice, and stained with propidium iodine (PI) at a final concentration of 10 μg/mL for 10 min. The samples were then subjected to a Guava^®^ EasyCyte™ system (Luminex, Corp., Austin, TX, US) for flow cytometric analysis. PI fluorescence was recorded using the RED-B channel (695/50 nm).

### 2.7 Biofilm formation assay

To investigate the effect of *fliA* deletion on biofilm formation by *E. coli* BW25113, crystal violet (CV) staining and confocal laser scanning microscopy (CLSM) were performed on *E. coli* BW25113, JW1907, and ReJW1907.

#### 2.7.1 CV staining assay

CV staining was performed according to the method described by Bian et al. (2022) with modifications. Briefly, 100 μL of *E. coli* culture (10^4^ CFU/mL) in LB broth was added to each well of a 96-well plate and cultured for 24 h at 37°C. Equal volumes of sterile LB broth were added to the plates as blank controls. Subsequently, the cultures were removed from each well, and unadhered *E. coli* cells were washed with 0.1 M PBS buffer. For biofilm fixation, 200 μL of 95% methanol was added to each well for 15 min at 25 ± 2°C. After fixation, the methanol was removed, and the plate was dried on filter paper for 10 min at 25 ± 2°C. Next, 200 μL of 1% CV solution was added to each well and stained for 10 min. The plate was rinsed thrice with sterile ddH_2_O and thoroughly dried in an incubator at 37°C for 15 min. Finally, 200 μL of 33% glacial acetic acid was added to dissolve the CV for 10 min at 37°C, and the plate was instantly subjected to a SpectraMax^®^ Mini multifunctional microplate reader (Molecular Devices, Shanghai, China). The absorbance at 595 nm of each well was measured thrice. Five biological replicates were included for each *E. coli* strain.

#### 2.7.2 CLSM

CLSM was used to observe biofilm formation on a non-biological surface by the three *E. coli* strains, according to the protocol described by Bian et al. (2022), with modifications. Glass-bottom confocal dishes (Cat# BS-20-GJM, Biosharp, China) were pre-equilibrated with 3 mL of sterile LB broth at 37°C for 15 min. After removing the LB broth, 500 μL of *E. coli* culture (10^4^ CFU/mL) in LB broth was added to the glass bottom and statically incubated for 2 h at 37°C to settle *E. coli* cells. Next, 2 mL of sterile LB broth was gently added to the confocal dishes before a 24-h incubation at 37°C. The cultures were removed from the dishes, and unadhered cells were gently washed thrice with 0.1 M PBS buffer. Biofilms on the confocal dishes were stained for 30 min with 100 μL of 10 μM 4′,6-diamidino-2-phenylindole (DAPI) at 25 ± 2°C. Finally, the dishes were subjected to a Leica STELLARIS 5 CLSM (Leica, Germany) for 2D and 3D imaging. The CLSM images were analyzed by LAS X software (version 3.7.4. 23463; Leica, Germany). CLSM Z-axis stacks were collected through the entire depth of DAPI-stained *E. coli* biofilms and were reconstructed in maximum projection images.

### 2.8 RNA-sequencing and transcriptomic analysis

To investigate the regulatory role of FliA in the antibacterial mechanism of plantaricin BM-1 against *E. coli*, RNA-sequencing (RNA-seq) was performed on *E. coli* BW25113 and JW1907. *E. coli* strains were cultured in LB broth for 12 h, as described in section 2.1. Three biological replicates were used for both *E. coli* strains. Bacterial samples were collected through centrifugation (8,000 rpm, 4°C, 10 min), washed thrice with 0.1 M PBS, subjected to liquid nitrogen for 10 min, and preserved at −80°C.

Samples were sent to Shanghai Majorbio Bio-pharm Technology Co., Ltd for subsequent RNA-seq. Library preparation was performed using a TruSeq™ Stranded Total RNA Library Prep Kit (Illumina, Inc., San Diego, CA, US). Briefly, total RNA was extracted from both *E. coli* cells, and ribosomal RNA was removed. The enriched mRNA was randomly fragmented into approximately 200 bp pieces, which served as templates for reverse transcription to synthesize cDNAs. The sticky ends of double-stranded cDNAs were supplemented by End repair Mix to become blunt ends, and the adapter was added to the 3′ end of cDNA. Before PCR enrichment, the second strand was digested with uracil-N-glycosylase (UNG). The library was enriched and subjected to Illumina NovaSeq X Plus platform (Illumina, Inc., San Diego, CA, US) for sequencing. Quality control (QC) was performed on raw data files, and the sequences of the clean reads from each sample were aligned with the reference genome of *E. coli* K-12 BW25113 (NCBI Assembly No. GCF_000750555.1). The RSEM software (Version 1.1.3) was used to quantitatively analyze gene expression levels. Normalization of gene expression was performed using transcripts per million (TPM), according to the following formula:


$${\mathrm{TPM}}_{i}=\frac{{\mathrm{Counts}}_{i}\times 10^{6}}{{\mathrm{Length}}_{i}\times \sum_{i}^{m}\frac{{\mathrm{Counts}}_{i}}{{\mathrm{Length}}_{i}}}$$

where TPM*_*i*_* represents the calculated TPM value of gene (*i*); Counts*_*i*_* represents the mapped read counts for gene (*i*); Length*_*i*_* represents the length (kb) of gene (*i*); $\sum_{i}^{m}\frac{{\mathrm{Counts}}_{i}}{{\mathrm{Length}}_{i}}$ represents the sum of $\frac{{\mathrm{Counts}}_{i}}{{\mathrm{Length}}_{i}}$ for all genes.

Differential analysis was performed using DESeq2 software (Version 1.42.0), and differentially expressed genes (DEGs) were screened with the following thresholds: *p*-adjust < 0.05; |log_2_Fold Change (FC)| ≥ 1. Functional annotation analysis of the DEGs was conducted using the Gene Ontology (GO) database. The Kyoto Encyclopedia of Genes and Genomes (KEGG) database was used to analyze the functions of biological pathways associated with the DEGs.

### 2.9 Reverse transcription quantitative PCR

Supplementary Table S1 lists all primers used for reverse transcription quantitative PCR (RT-qPCR), and the housekeeping gene GADPH (gapA) was used as the internal control. Total RNA of E. coli BW25113 and JW1907 was extracted using a RNAprep Pure Cell/Bacteria Kit (Tiangen Biotech, China). An RT-qPCR system (25 μL) was established by a Fastking one step RT-qPCR kit (Tiangen Biotech, China): 2 × Fastking RT-qPCR buffer (SYBR Green), 12.5 μL; 25 × Enzyme mix, 1 μL; forward and reverse primers (10 μM), 0.62 μL; RNA template, 0.5 μL; RNase-free water to a total volume of 25 μL. Amplification was performed using the LightCycler^®^ 96 real-time PCR system (Roche, Swiss); the RT-qPCR conditions are presented in Supplementary Table S2. Relative gene expression was calculated using the 2^–ΔΔCt^ method. All experiments were performed in triplicate.

### 2.10 Statistical analysis

All statistical analyses were performed using SPSS Statistics software V27.0.1.0 (IBM, corp., Armonk, NY, United States). Data are presented as mean ± standard deviation (SD). Mean comparisons were performed by one-way analysis of variance (ANOVA), followed by Dunnett’s *t*-test or Duncan’s test. The significance level was set to 0.05. All growth curves and bar charts were plotted using GraphPad Prism software version 10.4.1 (627) (GraphPad Software, Boston, MA, United States).

## 3 Results

### 3.1 Validation of *fliA*-complemented transformant *Escherichia coli* ReJW1907

**FIGURE 1:**
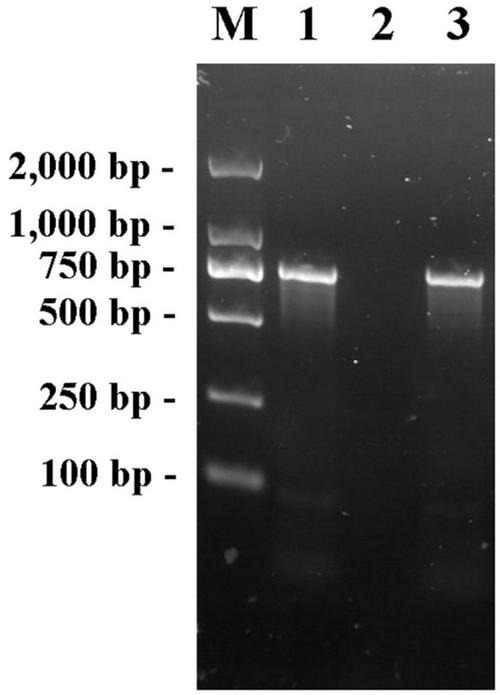
PCR products of primer pair fliA-Red-F/R in the genome of *E. coli* BW25113, JW1907 and ReJW1907. Lane M: Takara DL2,000 DNA Marker; Lane 1: Product amplified from *E. coli* BW25113; lane 2: Product amplified from *E. coli* JW1907; and lane 3: Product amplified from *E. coli* ReJW1907.

The fliA-Red-F/R primer pair was used to amplify the genomes of *E. coli* K12, JW1907, and positive transformants using PCR. The electrophoretogram shown in Figure 1 confirms that the *fliA* fragment (760 bp) was amplified from the genomes of *E. coli* BW25113 and the positive transformant, not *E. coli* JW1907, showing the successful transformation of the *fliA* gene into *E. coli* JW1907. The *fliA*-complemented transformant was renamed *E. coli* ReJW1907.

### 3.2 FilA regulates the antibacterial activity of plantaricin BM-1 against *Escherichia coli* BW25113

**FIGURE 2:**
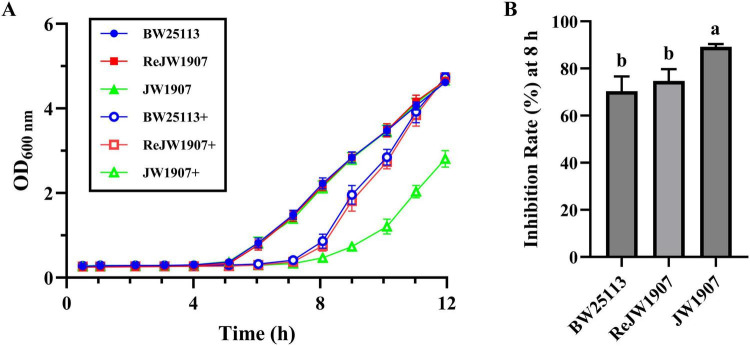
Effect of *fliA* deletion on the antibacterial activity of plantaricin BM-1 against *E. coli* BW25113. **(A)** Bacterial growth curves of *E. coli* BW25113, JW1907, and ReJW1907 treated with or without plantaricin BM-1. + represents plantaricin BM-1 treatment (3.75 mg/mL). Initial cell concentration of *E. coli* was adjusted to 10^4^ CFU/mL. **(B)** Inhibition rate (%) of plantaricin BM-1 (3.75 mg/mL) in *E. coli* BW25113, ReJW1907 and JW1907 after 8 h of culture. Results are presented as the mean ± SD, n = 3/group; mean comparisons were performed using one-way ANOVA, followed by Duncan’s test; a-b: different letters represent significant differences (*p* < 0.05) between groups.

The untreated *E.coli* BW25113, ReJW1907, and JW1907 cells in the control group exhibited similar growth curves (Figure 2A), entering the exponential phase within 5 h of cultures, with OD_600__nm_ values reaching 4.619 ± 0.086, 4.659 ± 0.049, and 4.660 ± 0.085 by 12 h, respectively. Significant delays in entering the exponential phase were observed in all three *E. coli* strains following plantaricin BM-1 treatment (3.75 mg/mL), demonstrating its antibacterial activity. Plantaricin-treated *E. coli* BW25113 and ReJW1907 entered the exponential phase after 7 h, with OD_600__nm_ values reaching 4.738 ± 0.108 and 4.728 ± 0.098, respectively. In comparison, plantaricin-treated *E. coli* JW1907 entered the exponential phase later, by 8 h, with a peak OD_600__nm_ of 2.810 ± 0.196 at 12 h.

The inhibition rate (I%) after 8 h of culture in plantaricin BM-1 (3.75 mg/mL)-treated *E. coli* JW1907 (89.22 ± 1.13%) was significantly higher (*p* < 0.01) than that of *E. coli* BW25133 (70.36 ± 6.30%) and *E. coli* ReJW1907 (74.75 ± 4.99%; Figure 2B). No significant difference was observed between the I% of *E. coli* BW25133 (70.36 ± 6.30%) and ReJW1907 (74.75 ± 4.99%; Figure 2B).

### 3.3 Cell morphology of plantaricin BM-1-treated *Escherichia coli* cells

**FIGURE 3:**
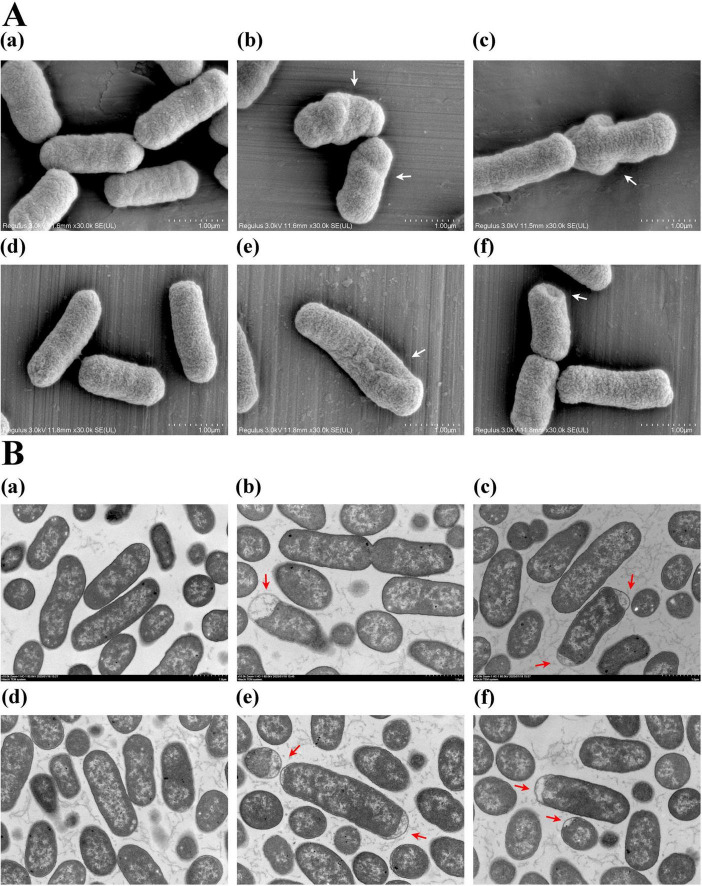
Effect of plantaricin BM-1 on *E. coli* cell morphology. **(A)** SEM images (magnification: 30,000 ×) showing the external condition of *E. coli* cells treated with or without plantaricin BM-1 (3.75 mg/mL); **(B)** TEM images (magnification: 10,000 ×) displaying the internal condition of *E. coli* cells treated with or without plantaricin BM-1 (3.75 mg/mL); (a) *E. coli* BW25113 control group; (b) *E. coli* BW25113 treated with plantaricin BM-1 for 8 h; (c) *E. coli* BW25113 treated with plantaricin BM-1 for 12 h; (d) *E. coli* JW1907 control group; (e) *E. coli* JW1907 treated with plantaricin BM-1 for 8 h; (f) *E. coli* JW1907 treated with plantaricin BM-1 for 12 h. White arrows: cell distortion and collapse; red arrows: plasmolysis.

To investigate the effect of plantaricin BM-1 on *E. coli* cell morphology, SEM and TEM analyses were performed on *E. coli* BW25113 and JW1907 following treatment with plantaricin BM-1 (3.75 mg/mL) for 8 or 12 h. *E. coli* BW25113 and JW1907 cells in the untreated groups appeared complete, with a full cylindrical shape with blunt ends (Figure 3Aa,d) and a dense and uniform internal structure with clear, intact edges (Figure 3Ba,d). This indicated that *fliA* deletion alone did not lead to visible cell damage in *E. coli*. However, after 8 or 12 h of plantaricin BM-1 treatment, the external shape of *E. coli* BW25113 and JW1907 cells shrunk significantly with distortion and collapse (Figure 3Ab,c,e,f); slight plasmolysis was observed inside both *E. coli* strains (Figure 3Bb,c,e,f). Notably, no visible fractures or pore formation were observed on the cell surfaces.

### 3.4 Effect of plantaricin BM-1 on the cell membrane integrity of *Escherichia coli* BW25113

**FIGURE 4:**
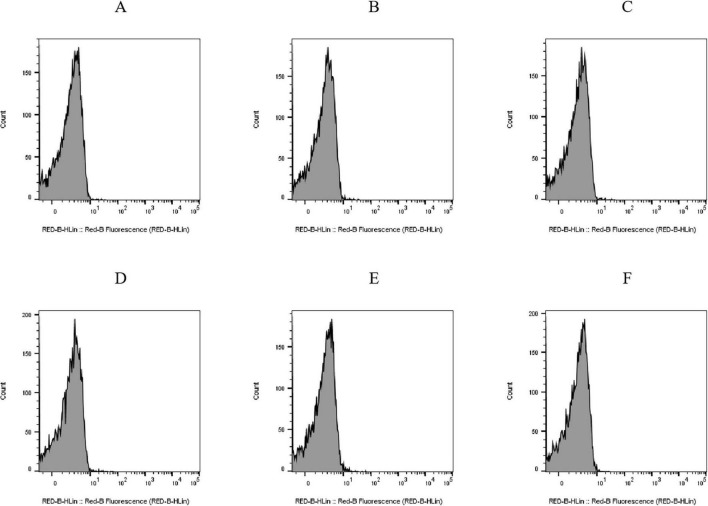
Effect of plantaricin BM-1 (3.75 mg/mL) on the cell membrane integrity of *E. coli* cells. Y-axis: cell counts; X-axis: PI fluorescence intensity. **(A)** *E. coli* BW25113 control group; **(B)** *E. coli* BW25113 treated with plantaricin BM-1 for 8 h; **(C)** *E. coli* BW25113 treated with plantaricin BM-1 for 12 h; **(D)** *E. coli* JW1907 control group; **(E)** *E. coli* JW1907 treated with plantaricin BM-1 for 8 h; and **(F)** *E. coli* JW1907 treated with plantaricin BM-1 for 12 h. PI fluorescence was recorded through the RED-B channel (695/50 nm).

PI is a fluorescent molecule that only penetrates impaired cells and binds DNA (Li et al., 2013; Crowley et al., 2016). Histograms of cell counts versus PI fluorescence intensity for the untreated *E. coli* BW25113 (Figure 4A) and JW1907 (Figure 4D) exhibited single peaks with cell counts corresponding to fluorescence distributed below 10^1^. The PI uptakes by *E. coli* BW25113 (Figures 4A–C) and JW1907 (Figures 4D–F) did not increase following 8 h or 12 h of plantaricin BM-1 (3.75 mg/mL) treatment compared to the untreated control group. This showed that the membrane integrity of both treated *E. coli* strains was not significantly damaged.

### 3.5 *fliA* deletion affects *Escherichia coli* BW25113 biofilm formation

**FIGURE 5:**
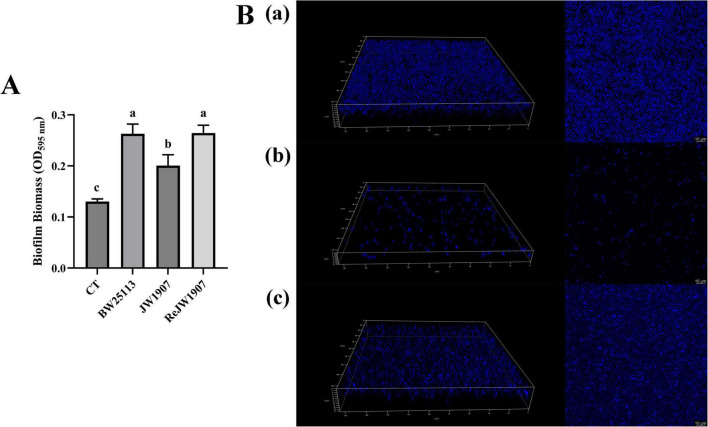
Biofilm formation of *E. coli* BW25113, JW1907, and ReJW1907. **(A)** Biofilm production of *E. coli* strains after 24 h; CT: 100 μL sterile LB broth (blank control); results are presented as the mean ± SD; *n* = 5/group; mean comparisons were performed using one-way ANOVA, followed by Duncan’s test; a-c: different letters represent significant differences (*p* < 0.05) between groups; **(B)** CLSM 3D and 2D images (magnification: 63 ×) of biofilms formed after 24 h by *E. coli* BW25113 (a), JW1907 (b) and ReJW1907 (c).

The biofilm production of *E. coli* BW25113, JW1907, and ReJW1907 was quantified by CV staining after 24-h of incubation. All three *E. coli* strains formed biofilms, with biomasses significantly higher (*p* < 0.05) than that of the blank control (OD_595__nm_ = 0.130 ± 0.005; Figure 5A). The biofilm biomass produced by *E. coli* JW1907 (OD_595_ = 0.227 ± 0.04) was significantly lower (*p* < 0.05) than those of *E. coli* BW25113 (OD_595__nm_ = 0.343 ± 0.056) and ReJW1907 (OD_595__nm_ 0.358 ± 0.027); however, no significant difference was observed between *E. coli* BW25113 and ReJW1907.

The 3D and 2D CLSM images of the biofilms revealed that after 24 h, *E. coli* BW25113 (Figure 5Ba) and ReJW1907 (Figure 5Bc) formed dense, evenly distributed, multilayered biofilms on the glass bottom. In contrast, *E. coli* JW1907 did not form a significant biofilm, with degeneration of the biofilm structure and cell adhesion observed, resulting in reduced biofilm biomass and thickness (Figure 5Bb).

### 3.6 Transcriptomic analysis of *Escherichia coli* BW25113 and JW1907

#### 3.6.1 Screening differentially expressed genes

**FIGURE 6:**
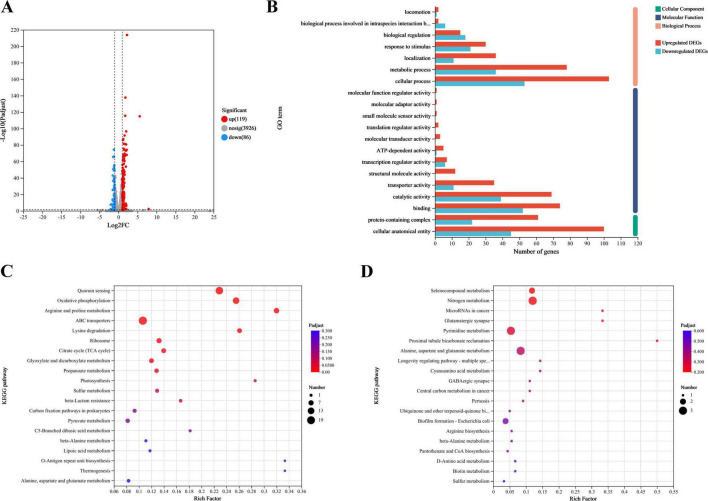
Transcriptomic analysis of *E. coli* BW25113 and JW1907. **(A)** Differences in gene expression between *E. coli* BW25113 and JW1907. Each dot represents one gene; red: up-regulated in *E. coli* JW1907, blue: down-regulated in *E. coli* JW1907; gray: no significant differences in expression; and hatched lines: threshold for screening DEGs (*p*-adjust < 0.05; |log_2_FC| ≥ 1; **(B)** GO functional annotation analysis of DEGs; **(C)** KEGG pathways associated with the 119 up-regulated DEGs; **(D)** KEGG pathways associated with the 86 down-regulated DEGs; top 20 KEGG pathways in DEG enrichment were used to plot bobble charts; *n* = 3/group.

Transcriptomic analysis identified 4,131 known genes from the reference genome with annotations, with gene expression detected for 4094. DEGs were screened to investigate the function of FliA in regulating the antibacterial mechanism of plantaricin BM-1 against *E. coli* BW25113. A total of 205 DEGs (fold change ≥ 2, *p*-adjust < 0.05) were identified in the *E. coli* BW25113 versus JW1907 comparison, of which 119 were up-regulated and 86 down-regulated significantly (*p* < 0.05; Figure 6A).

#### 3.6.2 Functional annotation and enrichment analysis of DEGs

##### 3.6.2.1 GO Functional annotation analysis of DEGs

Using GO functional annotation analysis, the 205 DEGs were classified into cellular components (CC), molecular functions (MF), and biological processes (BP) (Figure 6B). Among them, 158 DEGs were annotated to CC as a cellular anatomical entity (GO:0110165) or protein-containing complex (GO:0032991); 169 DEGs were annotated to MF, with the top three terms being binding (GO:0005488), catalytic activity (GO:0003824) and transporter activity (GO:0005215); 170 DEGs were annotated to BP, and the top three terms were cellular process (GO:0009987), metabolic process (GO:0008152), and response to stimulus (GO:0050896).

##### 3.6.2.2 KEGG pathway enrichment analysis of DEGs

The bobble charts in Figure 6 show the KEGG pathways enriched in the 119 upregulated and 86 downregulated DEGs. The up-regulated DEGs were enriched in 47 KEGG pathways; the top five (*p*-adjust < 0.001) were quorum sensing (map02024), oxidative phosphorylation (map00190), arginine and proline metabolism (map00330), ABC transporters (map02010), and lysine degradation (map00310) (Figure 6C). 53 upregulated DEGs that significantly enriched in the top five KEGG pathways were listed in Supplementary Material 2. Although 86 downregulated DEGs were enriched in 25 KEGG pathways with no statistical significance, the top three pathways were selenocompound metabolism (map00450), nitrogen metabolism (map00910), and microRNAs in cancer (map05206) (Figure 6D).

Among the 205 DEGs, we discovered 4 DEGs (*fliZ*, *wza*, *lsrR*, and *pgaA*) that are enriched in the Biofilm Formation-*Escherichia coli* pathway (map02026) (Table 2). Notably, the results showed that 8 up-regulated DEGs (*lsrKRBDCAFG*) are significantly enriched in the LuxS/AI-2 quorum sensing (QS) system (map02024; *Escherichia coli*; *p*-adjust < 0.001) (Table 3), indicating the overall up-regulation of this particular QS system.

**TABLE 2:** Changes in expression of biofilm formation-*Escherichia coli* pathway (map02026)-associated DEGs.

Gene ID	Gene name	Gene description	Fold change (JW1907/BW25113)	*p*-adjust
BW25113_1921	*fliZ*	RpoS antagonist; putative regulator of FliA activity	47.344	1.2827780081E-115
BW25113_1512	*lsrR*	*lsr* operon transcriptional repressor	2.018	1.13334861245E-30
BW25113_0983	*gfcE/wza*	Polysaccharide biosynthesis/export protein	0.497	1.63877118442E-13
BW25113_1024	*pgaA*	Putative O-antigen capsule outer membrane auxillary protein export channel	0.44	0.0288236679955

**TABLE 3:** Changes in the expression of the LuxS/AI-2 QS system-associated DEGs.

Gene ID	Gene name	Gene description	Fold change (JW1907/BW25113)	*p*-adjust
BW25113_1511	*lsrK*	Autoinducer-2 (AI-2) kinase	2.057	2.86236434718E-30
BW25113_1512	*lsrR*	*lsr* operon transcriptional repressor	2.018	1.13334861245E-30
BW25113_1513	*lsrA*	Autoinducer 2 import ATP-binding protein	2.198	1.14677258858E-27
BW25113_1514	*lsrC*	Autoinducer 2 import system permease protein	2.53	5.41006977077E-24
BW25113_1515	*lsrD*	autoinducer 2 import system permease protein	2.801	1.09439795795E-20
BW25113_1516	*lsrB*	Autoinducer 2-binding protein	2.472	1.72157992622E-31
BW25113_1517	*lsrF*	Putative autoinducer-2 (AI-2) aldolase	2.974	3.02408517728E-48
BW25113_1518	*lsrG*	Autoinducer-2 (AI-2) degrading protein LsrG	2.52	2.48407172299E-13

### 3.7 RT-qPCR validation of LuxS/AI-2 QS system-associated DEGs

**FIGURE 7:**
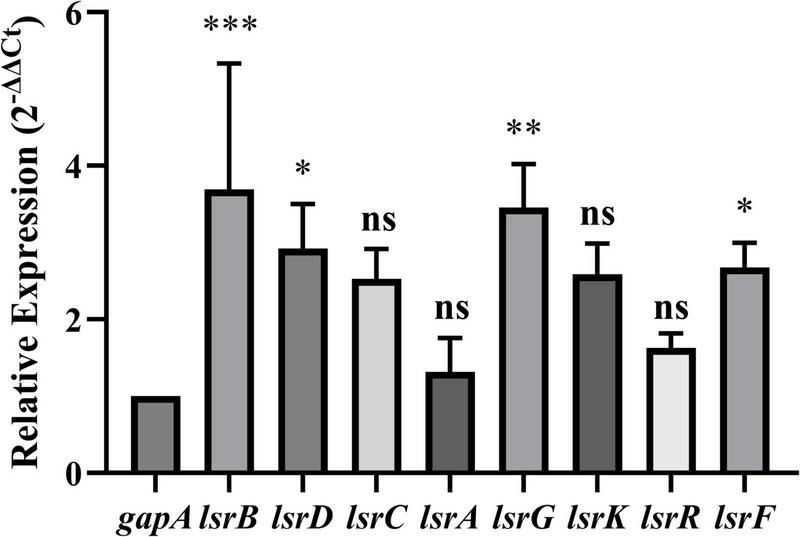
Relative expression of LuxS/AI-2 quorum sensing system-related differentially expressed genes (DEGs) in *E. coli* JW1907. GADPH (*gapA*) served as the internal control. Data are presented as the mean ± SD; *n* = 3/group; mean comparisons were performed using one-way ANOVA, followed by Dunnett’s *t*-test; *p < 0.05, **p < 0.01, and ***p < 0.001 between the relative expression of each DEG and the internal control; ns: no significant difference.

To further evaluate the expression levels of the eight DEGs (*lsrKRBDCAFG*) associated with the LuxS/AI-2 QS system, RT-qPCR was performed on the total RNA extracted from *E. coli* BW25113 and JW1907. The relative expressions of *lsrB*, *lsrD*, *lsrG*, and *lsrF* were significantly up-regulated by 3.693 ± 1. 641-, 2.924 ± 0. 578-, 3.456 ± 0. 570-, and 2.676 ± 0.320-fold, compared with *gapA*, respectively (*p*-adjust < 0.05) (Figure 7). The relative expressions of *lsrC*, *lsrA*, *lsrK*, and *lsrR* were also up-regulated by 2.529 ± 0. 391-, 1.315 ± 0. 444-, 2.587 ± 0. 402-, and 1.628 ± 0.188-fold compared with *gapA*; however, statistical significance was not reached.

### 3.8 LuxS/AI-2 QS system-associated DEGs regulate the antibacterial activity of plantaricin BM-1 against *Escherichia coli* BW25113

**FIGURE 8:**
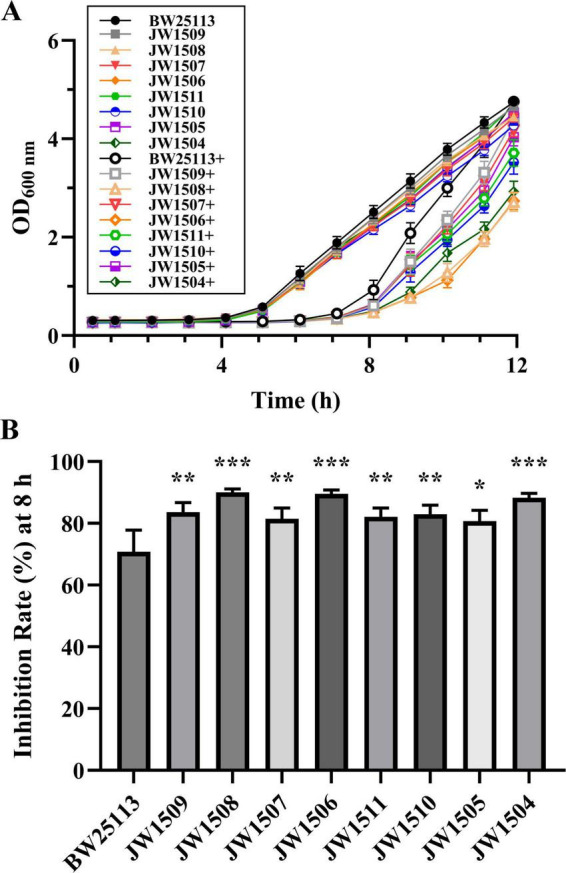
Effect of the LuxS/AI-2 QS system-related DEG deletions on the antibacterial activity of plantaricin BM-1 against *E. coli* BW25113. **(A)** Bacterial growth curves of *E. coli* BW25113, JW1509, JW1508, JW1507, JW1506, JW1511, JW1510, JW1505, and JW1504 with or without plantaricin BM-1 treatment. + represents plantaricin BM-1 treatment (3.75 mg/mL). Initial cell density of *E. coli* was adjusted to 10^4^ CFU/mL; **(B)** inhibition rate (%) of plantaricin BM-1 (3.75 mg/mL) against *E. coli* BW25113 and its LuxS/AI-2 QS pathway-associated gene null mutants after 8 h of culturing. Results are presented as the mean ± SD, *n* = 3/group; mean comparisons were performed using one-way ANOVA, followed by Dunnett’s *t*-test; *p < 0.05, **p < 0.01, and ***p < 0.001 between the single-gene knock-out mutant and *E. coli* BW25113.

To determine whether the DEGs associated with the LuxS/AI-2 QS system contributed to the antibacterial mechanism of plantaricin BM-1 against *E. coli* BW25113, bacterial growth curves were constructed following treatment of *E. coli* BW25113 and 8 single-gene knockout mutants (*E. coli* JW1509, JW1508, JW1507, JW1506, JW1511, JW1510, JW1505, and JW1504) with plantaricin BM-1 (3.75 mg/mL) for 12 h (Figure 8A).

Untreated *E. coli* BW25113 and the eight mutants in the control group entered the exponential phase after 5 h of culture, with OD_600__nm_ values peaking between 4.275 ± 0.079 and 4.749 ± 0.069 after 12 h. The logarithmic growth of the nine *E. coli* strains treated with plantaricin BM-1 (3.75 mg/mL) was delayed, with the OD_600__nm_ of treated *E. coli* BW25113 exhibiting a steeper increase than those of the eight mutants after 8 h of culturing. Moreover, the I% of plantaricin BM-1 (3.75 mg/mL) for *E. coli* BW25113 (70.77 ± 7.01%) was significantly lower (*p* < 0.05) than that for any mutant (from 80.68 ± 3.50% to 90.07 ± 1.08%) after 8 h (Figure 8B).

### 3.9 LuxS/AI-2 QS system-associated DEGs regulate *Escherichia coli* BW25113 biofilm formation

To further investigate the relationship between the LuxS/AI-2 QS system and biofilm formation in *E. coli* K-12, CV staining was performed on *E. coli* BW25113 and eight LuxS/AI-2 QS system-related gene mutants, including *E. coli* JW1509, JW1508, JW1507, JW1506, JW1511, JW1510, JW1505, and JW1504.

**FIGURE 9:**
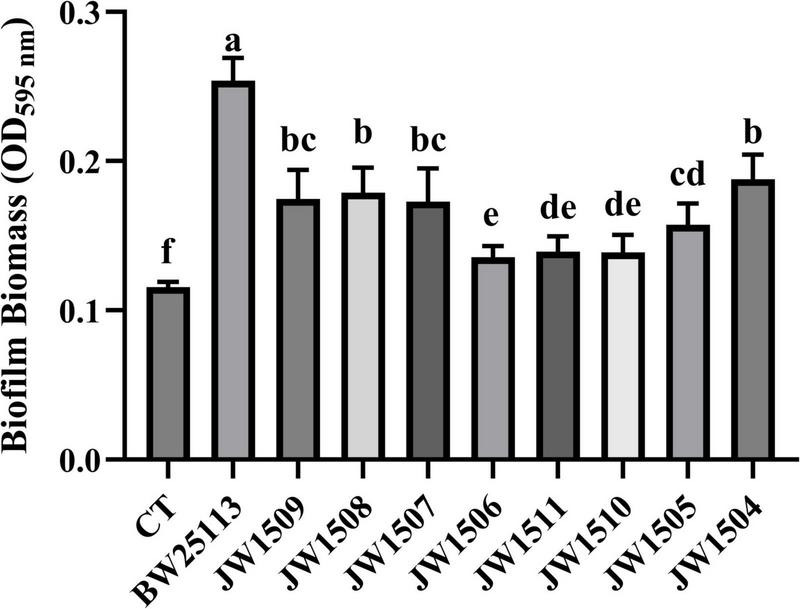
Biofilm production by *E. coli* BW25113 and LuxS/AI-2 QS pathway-associated gene null mutants. CT: 100 μL sterile LB broth (blank control). Results are presented as the mean ± SD; *n* = 5/group; mean comparisons were performed using one-way ANOVA, followed by Duncan’s test. a-f: different letters represent significant differences (*p* < 0.05) between groups.

All *E. coli* strains formed biofilms, with biomasses that were significantly higher (*p* < 0.05) than that of the blank control (OD_595__nm_ = 0.116 ± 0.003; Figure 9). After 24-h incubation, *E. coli* BW25113 produced significantly more (*p* < 0.05) biofilm biomass (OD_595__nm_ = 0.254 ± 0.014) than the eight LuxS/AI-2 QS system-related gene mutants (from 0.135 ± 0.007 to 0.188 ± 0.015), showing a regulatory role of the LuxS/AI-2 QS system in *E. coli* biofilm formation.

## 4 Discussion

In this study, we confirmed that FliA (σ^28^) is involved in the bacteriostasis of plantaricin BM-1 against *E. coli* K-12 BW25113. The plantaricin treatment leads to changes in the cell morphology of *E. coli* BW25113 and JW1907, but caused almost no damage to cell membrane integrity. Also, we did not observe visible changes in *E. coli* BW25113 morphology after deleting *fliA*. Therefore, we hypothesized that FliA may regulate antibacterial activity through other possible means. It is later discovered that FliA regulates *E. coli* biofilm formation and the LuxS/AI-2 QS system-associated gene expression. Using growth curve analysis and CV staining assay, we further validated the regulatory role of the LuxS/AI-2 QS system-associated DEGs in *E. coli* biofilm formation, and their influence on plantaricin BM-1 bacteriostasis.

Anti-biofilm activity against *E. coli* has been reported for several LAB bacteriocins (Qiao et al., 2021; Kim et al., 2019; Luo et al., 2020; Xin et al., 2023), including plantaricin BM-1. Wang et al. (2021) first reported the anti-biofilm activity of plantaricin BM-1 against *E. coli* BW25113, and discovered that outer membrane channel protein TolC regulates the bacteriostasis of the plantaricin through biofilm modulation. Bian et al. (2022) and Wang et al. (2025) discovered that after the deletion of *rcsC* (encoding the Rcs phosphorelay component RcsC) or *potF* (enconding the putrescine ABC transporter substrate-binding protein PotF), the sensitivity of *E. coli* K-12 to plantaricin BM-1 increases with a significant decrease in biofilm formation, indicating a high correlation between the bacteriostasis of the plantaricin and *E. coli* biofilm formation. Besides, previous evidence revealed a connection between flagellum-associated FliA and biofilm formation in *E. coli*. Wood et al. (2006) observed a dramatic decrease in *E. coli* K-12 MG1655 biofilm formation after *fliA* deletion. Notably, Buck et al. (2021) discovered that FliA overexpression in *E. coli* PHL628 significantly increases biofilm production after 48 h at 37°C. Therefore, we hypothesized that FliA regulates antibacterial activity through biofilm modulation. The CV staining and CLSM results confirmed that *E. coli* JW1907 could not form a dense or structured biofilm compared to *E. coli* BW25113 and ReJW1907, indicating that FliA regulates *E. coli* biofilm formation.

To further investigate the regulatory role of FliA, transcriptomic analysis was performed on *E. coli* BW25113 and JW1907. KEGG pathway enrichment analysis of the identified DEGs revealed 4 DEGs (*fliZ*, *wza*, *lsrR*, and *pgaA*) that are enriched in the biofilm formation-*Escherichia coli* pathway (map02026). *fliZ* encodes a global regulatory protein FliZ, which is regulated by the flagellar master regulator FlhDC (Pesavento and Hengge, 2012). FliZ binds to specific DNA regions to repress the expression of numerous genes under the control of sigma factor RpoS (σ^S^), including *csgD*, which encodes the biofilm master regulator CsgD (Mika and Hengge, 2014). Notably, in this study, the expression of *fliZ* was significantly upregulated (*p*-adjust < 0.05) in *E. coli* JW1907. Li et al. (2007) discovered that the expression of the biofilm-associated gene *wza* (*gfcE*), responsible for colanic acid biosynthesis, was significantly upregulated in the Δ*lsrR* strains of *E. coli* K-12 W3110. After deleting *fliA* in *E. coli* BW25113, we noticed that *lsrR* was significantly upregulated, whereas *wza*/*gfcE* was downregulated, indicating a negative regulation of Wza by LsrR. *pgaA* encodes the adhensin poly-beta-1,6-N-acetyl-D-glucosamine synthesis-related protein PgaA, which is necessary for biofilm formation (Itoh et al., 2008). A previous study by Gong et al. (2021) demonstrated that the deletion of *pga* genes inhibits the biofilm production of *E. coli* K-12 MG1655. Kang et al. (2018) discovered that gallic acid inhibits the biofilm formation of *E. coli* ATCC 25922 through downregulating *pgaA* expression. In this study, *pgaA* was significantly downregulated in *E. coli* JW1907. These four DEGs indicate that the deletion of *fliA* triggered changes in the expression of genes the biofilm formation pathway, which is consistent with the CV staining and CLSM results.

Through further analysis of the 53 up-regulated DEGs, we discovered that *lsrR* and the other 7 *lsr* genes (*lsrKBDCAFG*), which express the LuxS/AI-2 QS system, were all significantly up-regulated (*p*-adjust < 0.05) after *fliA* deletion. This result indicates a regulation of this QS system by FliA, which we attribute to *E. coli* responding to the potential stress of *fliA* deletion by up-regulating the expression of genes promoting biofilm formation. Although so far, no evidence has shown that sigma factor FliA can activate the transcription of genes associated with the LuxS/AI-2 QS system directly, yet previous studies have revealed a connection between this QS system and FliA in *E. coli*. Kim et al. (2010) and Sperandio et al. (2001) both discovered that *fliA* is down-regulated in the Δ*luxS* mutant of enterohemorrhagic *E. coli* O157:H7 through the transcriptomic analysis. Jani et al. (2017) demonstrated that the LuxS/AI-2 QS system regulates the biofilm formation in *E. coli*. They observed a significant decrease in the biofilm formation by *E. coli* RP437Δ*lsrB*, Δ*lsrR*, Δ*lsrK*, Δ*lsrC*, Δ*lsrG*, and Δ*luxS* strains, compared with the wild-type *E. coli* RP437, which indicates the lack of LuxS/AI-2 QS system components decreases *E. coli* biofilm production. Therefore, we hypothesized that FliA regulates *E. coli* biofilm formation through the LuxS/AI-2 QS system, thus regulating the bacteriostasis of plantaricin BM-1 against *E. coli*.

Consistent with this assumption, the growth curve assay revealed that the LuxS/AI-2 QS system regulates the antibacterial activity of plantaricin BM-1 against *E. coli* BW25113, and CV staining results later confirmed the regulation of *E. coli* biofilm production by this QS system. As the up-regulation of 8 *lsr* genes is a direct consequence of *fliA* deletion in this study, it can be logically inferred that FliA modulates biofilm formation through the LuxS/AI-2 QS system, regulating the antibacterial activity of plantaricin BM-1. These findings improve our understanding of the bacteriostatic mechanism of class IIa bacteriocins against gram-negative bacteria, and provide a theoretical basis for the development of new antibacterial strategies based on quorum-sensing interference.

Future studies are required to investigate whether a direct connection exists between FliA and the LuxS/AI-2 QS system, as well as the regulatory role of this QS system in the bacteriostasis of plantaricin BM-1 against *E. coli*. Also, in this study, we focus on validating the connection between the eight LuxS/AI-2 QS system-associated DEGs (*lsrKRBDCAFG*) with *E. coli* biofilm formation and the antibacterial activity of plantaricin BM-1, whereas the regulatory role of *fliZ*, *wza*, and *pgaA* in both aspects remains to be discussed.

## Data Availability

The datasets presented in this study can be found in online repositories. The names of the repository/repositories and accession number(s) can be found at: https://www.ncbi.nlm.nih.gov/, PRJNA1234102.
