# Supplementary material for: FliA regulates the antibacterial activity of plantaricin BM-1 against Escherichia coli K-12 through the LuxS/AI-2 quorum-sensing-mediated biofilm formation
Source: Front Microbiol. 2025 Jun 20;16:1606567. doi: 10.3389/fmicb.2025.1606567 (PMC12226578; doi:10.3389/fmicb.2025.1606567)
Supplement: Supplementary file 1 [file Data_Sheet_1.docx]

Supplementary Material

**Table S1** Primers used for RT-qPCR.

| **Primers name** | **Sequence (5'→3')** |
| --- | --- |
| GADPH-F | CTGGTGCGAAGAAAGTGGTTATG |
| GADPH-R | GGTGGTGCAGGAAGCGTTG |
| lsrB-F | TGCTGACCTGGGACTCTG |
| lsrB-R | CCACCAACATACCTCCTAACT |
| lsrD-F | GGCGATACTCCTGACCTTAC |
| lsrD-R | TCGGGAATCCACCAATAC |
| lsrC-F | GTGCTGGGCGGCATTAGT |
| lsrC-R | CGCAGGCGTCCATCAAAC |
| lsrA-F | CGAAAGATAATGCCACCC |
| lsrA-R | AGGAGATAAGCAGCACAGC |
| lsrG-F | GAACCACCTGGGCTCTGTA |
| lsrG-R | CCACTGCGTCTTCATCTTTAT |
| lsrF-F | TACCCATTGTTATTGCTG |
| lsrF-R | GCCTGTACGGCTTTCATC |
| lsrR-F | GCAATGTCAGCGGAGGATG |
| lsrR-R | CGGTGGCGTCGGTTCTTAT |
| lsrK-F | CCTAACCCAAGGCAACCAAGC |
| lsrK-R | TTCACAAGCGGCGGAACTCT |

**Table S2** RT-qPCR program.

| **Discription** | **Temperature (°C)** | **Time (s)** | **Acq. mode** | **Cycle** |
| --- | --- | --- | --- | --- |
| Reverse transcription | 50 | 1800 | none | 1 |
| Preincubation | 95 | 180 | none | 1 |
| 3-Step Amplification | 95 | 15 | none | 40 |
|  | 60 | 30 | none |  |
|  | 72 | 30 | Single |  |
| Melting | 95 | 10 | none | 1 |
|  | 65 | 60 | none |  |
|  | 97 | 1 | Continuous |  |
| Cooling | 37 | 30 | none | 1 |
